# What makes acute cholecystitis recur after removing the percutaneous cholecystostomy tube?

**DOI:** 10.1097/MD.0000000000028767

**Published:** 2022-02-04

**Authors:** Jun Heo, Min Kyu Jung, Chang Min Cho, Sang Yub Lee, Hun Kyu Ryeom, Jae Min Chun, Young Seok Han, Hyung Jun Kwon

**Affiliations:** a School of Medicine, Kyungpook National University, Daegu, the Republic of Korea; b Department of Internal Medicine, Kyungpook National University Hospital, Daegu, the Republic of Korea; c Department of Radiology, Kyungpook National University Hospital, Daegu, the Republic of Korea; d Department of Surgery, Kyungpook National University Hospital, Daegu, the Republic of Korea.

**Keywords:** acute, cholecystitis, drainage, endoscopic retrograde cholangiopancreatography, recurrence, sphincterotomy

## Abstract

Percutaneous transhepatic gallbladder drainage (PTGBD) is an alternative treatment option for acute cholecystitis. However, the disease may recur after PTGBD catheter removal. This study aimed to evaluate the role of endoscopic sphincterotomy and other risk factors in reducing the recurrence of cholecystitis.

We retrospectively analyzed data from 1088 patients who underwent PTGBD for cholecystitis at Kyungpook National University Hospital, Republic of Korea, between January 2011 and April 2018.

A total of 115 patients were enrolled in the study. The recurrence rate of cholecystitis was 17.4% (n = 20) during a median follow-up period of 1159 (range, 369–2774) days. Endoscopic biliary sphincterotomy did not significantly affect the recurrence rate of cholecystitis (*P* = .561). In multivariable analysis, cystic duct stones (*P* = .013) and PTGBD catheter migration before the prescheduled removal time (*P* = .002) were identified as independent risk factors for cholecystitis recurrence after PTGBD.

To reduce post-PTGBD recurrence in cholecystitis, caution must be exercised to avoid inadvertent dislodging of the PTGBD catheter. In cases of cholecystitis with cystic duct stones, cholecystectomy should be considered only after careful assessment of postoperative risks. Instead, transluminal endoscopic gallbladder drainage could represent a promising option for the prevention of recurrent cholecystitis.

## 1 Introduction

Cholecystectomy is the treatment of choice for acute cholecystitis. This procedure is a definitive treatment as it removes the gallbladder. However, many patients with cholecystitis are unsuitable candidates for surgery because of their comorbidities. Mortality after cholecystectomy is closely associated with comorbidities such as cirrhosis, heart failure, and renal failure. With the ever-increasing life expectancy and population aging globally, the prevalence of comorbidities associated with advanced age continues to increase as well. Hence, age >70 years is also a strong risk factor for death after cholecystectomy.^[1]^ Traditionally, percutaneous transhepatic gallbladder drainage (PTGBD) is considered an alternative treatment option for acute cholecystitis.^[2]^ This procedure is relatively safe for patients with comorbidities and has been validated as a bridge to cholecystectomy.^[3]^ However, there is a lack of evidence supporting the use of PTGBD as a definitive treatment option.

The PTGBD catheter was removed within 4 to 8 weeks after the procedure. However, disease recurrence remains the main concern associated with PTGBD catheter removal. Therefore, it is important to determine the factors affecting post-PTGBD recurrence in cholecystitis. The primary objective of this study was to determine the rate of recurrent cholecystitis after PTGBD and catheter removal. The secondary objective was to investigate the factors associated with the recurrence of cholecystitis following PTGBD and catheter removal.

## 2 Materials and methods

### 2.1 Patients

Patients treated with PTGBD at Kyungpook National University Hospital, Republic of Korea, between January 2011 and April 2018 were retrospectively reviewed. Of these, patients undergoing immediate PTGBD for acute cholecystitis with or without related cholelithiasis were deemed eligible for inclusion in the study. The exclusion criteria were as follows: planned cholecystectomy within 3 months after PTGBD, regardless of whether or not catheter removal was scheduled or conducted; a follow-up period of less than 1 year after PTGBD catheter removal; loss to follow-up before PTGBD catheter removal; no PTGBD catheter removal; secondary cholecystitis due to confirmed cholangitis—that is, reactive cholecystitis caused by, for example, common bile duct (CBD) stones or CBD strictures; malignancy-induced cholecystitis (eg, cholecystitis due to bile duct cancer invading the cystic duct); direct bile duct trauma; cholecystitis not confirmed to be calculous or acalculous, including conditions initially diagnosed as acalculous cholecystitis but proven to be calculous upon follow-up; and failure to meet the cholecystitis diagnostic criteria outlined by the Tokyo guidelines for acute cholangitis and cholecystitis.^[4]^ The study design was reviewed and approved by the Institutional Review Board of Kyungpook National University Hospital, Korea (approval no. 2020-01-017). The study protocol conformed to the ethical guidelines of the 1975 Declaration of Helsinki, as reflected by a priori approval from our institution's Human Research Committee.

### 2.2 Definition of acute cholecystitis

Patients were diagnosed with acute cholecystitis if they fulfilled the following criteria defined by the Tokyo guidelines for acute cholangitis and cholecystitis: local signs of inflammation (eg, Murphy's sign or right upper quadrant mass/pain/tenderness); systemic signs of inflammation, including fever, elevated C-reactive protein (CRP), or elevated white blood cell (WBC) count; and imaging findings characteristic of acute cholecystitis. Biliary disease is usually diagnosed on the basis of abdominal dynamic computed tomography. Other imaging modalities, such as abdominal ultrasonography, endoscopic ultrasonography, and magnetic resonance cholangiopancreatography, are also employed depending on the clinical needs. In this study, gallbladder sludge confirmed by imaging modalities was categorized as calculous cholecystitis.

### 2.3 PTGBD catheter insertion

When patients were diagnosed with acute cholecystitis, they were administered intravenous antibiotics (third-generation cephalosporins with/without metronidazole). Interventional radiologists then performed the PTGBD procedure. Under ultrasonographic guidance and fluoroscopic assistance, transhepatic gallbladder puncture was performed using a 15-cm, 21-gage AccuStick needle (Boston Scientific, Marlborough, MA). After serial dilation, an 8.5-French drainage catheter (Cook Medical, Bloomington, IN) was inserted into the gallbladder. Cholecystography was also performed during the same session after the drainage of the infected bile. Cystic duct patency and filling defects in the gallbladder were assessed, if necessary.

### 2.4 Follow-up

After treatment with antibiotics, patients were discharged with an indwelling PTGBD catheter and were followed up every 4 to 12 weeks until removal of the catheter. During outpatient follow-up, cholecystectomy was recommended if the patient was deemed suitable for surgery. However, for patients who were not fit for surgery, cystic duct patency was checked regularly until removal of the catheter.

### 2.5 PTGBD catheter removal

The decision to remove the PTGBD catheter was made according to the clinician's judgment after improvement of cholecystitis. The procedure could be performed in the outpatient department or interventional radiology department. Clamping of the catheter for 1 to 7 days before its removal was also left to the discretion of the treating clinician. Generally, the catheter was removed 8 to 12 weeks after insertion. In cases where catheter removal was performed in the radiology department, a follow-up cholangiogram was usually obtained before removal of the catheter.

### 2.6 Statistical analysis

All statistical analyses were performed using SPSS (version 22.0; IBM Corp., Armonk, NY). The results were analyzed using the *χ*^2^ test, Student *t* test, or Fisher exact test, as appropriate. A Cox proportional hazards model was used to examine the risk factors for disease recurrence. For all analyses, statistical significance was set at *P* < .05.

## 3 Results

### 3.1 Patient characteristics

**Figure 1:**
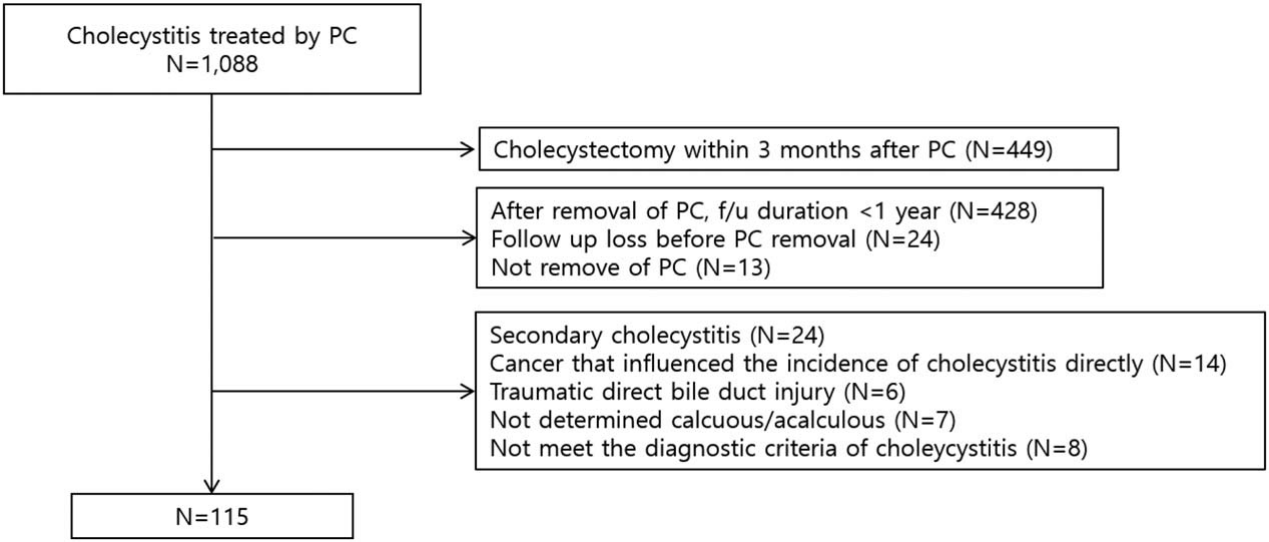
Flow chart of participants through study. PTGBD, percutaneous transhepatic gallbladder drainage.

A total of 1088 patients were reviewed during the study period, of whom 973 did not meet the inclusion criteria and 115 were enrolled in the study (Fig. 1). Sixty-eight patients (59.1%) were male, and the mean age was 73.2 ± 10.8 years. The mean Charlson Comorbidity Index (CCI) score was 4.5 ± 1.9. Calculous cholecystitis (n = 76, including 11 cases of gallbladder sludge) was more common than acalculous cholecystitis (66.1% vs 33.9%, respectively). Cystic duct stones were observed in nine cases (7.8%). There were 12 cases (10.4%) had perforated cholecystitis, all of which were recovered by PTGBD without surgical treatment. The mean duration of catheter indwelling was 151.4 ± 285.2 days (Table 1).

**Table 1:** Baseline demographic and clinical characteristics of the patients.

Characteristic	Patients who underwent PTGBD (n = 115)
Sex, male	68 (59.1)
Age, y	73.2 ± 10.8
Calculous	71 (66.1)
Gallstone	67 (56.5)
Size ≥1 cm	14 (12.2)
Size <0.5 cm	44 (38.3)
Solitary	22 (19.1)
Cystic duct stone	9 (7.8)
Sludge	24 (20.9)
Charlson Comorbidity Index score	4.6 ± 2.0
Perforation	12 (10.4)
CBD stone	21 (18.3)
Combined ERCP	41 (35.7)
Catheter indwelling time, days	151.4 ± 285.2
Duration of PTGBD ≥6 wk	89 (77.4)
Follow-up cholangiogram	67 (58.3)
Clamping of the PTGBD catheter before removal	41 (35.7)
PTGBD catheter migration	29 (25.2)
WBC count, cells/μL	13,196 ± 6225.0
CRP, mg/dL	15.0 ± 9.5
Bilirubin, serum, mg/dL	1.9 ± 2.3
Follow-up period, days	1212.6 ± 633.9 (range, 369–2774)

Data are presented as median (range), number (%), or mean ±  standard deviation. CBD = common bile duct, ERCP = endoscopic retrograde cholangiopancreatography, PTGBD = percutaneous transhepatic gallbladder drainage, SD = standard deviation, WBC = white blood cell.

### 3.2 PTGBD complications

The technical success rate of PTGBD was 99.1% (114/115 cases). In 1 patient, gallbladder drainage was achieved via the transperitoneal approach. No immediate procedure-related complications were noted, except for 2 cases of initial PTGBD catheter malfunction, which were corrected by catheter repositioning the following day. The main late PTGBD-associated complication was catheter migration, which occurred in 29 patients (25.2%). The interval between PTGBD insertion and migration was 227.2 ± 288.7 (mean ± standard deviation). Furthermore, 4 cases of recurrent catheter migration were observed despite reinsertion. There was only 1 case of late catheter malfunction that occurred 5 months after the initial insertion.

### 3.3 Recurrence of cholecystitis

The recurrence rate of cholecystitis was 17.4% (n = 20) during a median follow-up period of 1159 (range, 369–2774) days. The median interval between catheter removal and cholecystitis recurrence was 163.5 (range, 0–1276) days. In total, 41 patients underwent endoscopic biliary sphincterotomy because of CBD stones or strictures, with the median interval between PTGBD and biliary sphincterotomy being 108 (range, 0–1988) days. To be more precise, this procedure was conducted in 10 patients before PTGBD catheter insertion (2 cases during the same admission period and eight cases in the previous admission) with a median interval of 281.5 (range, 12–1536 days) and in 31 patients after PTGBD (18 cases during the same admission period and 13 cases during the next admission) with a median interval of 180.0 (range, 55–1988 days). Endoscopic biliary sphincterotomy had no significant effect on the recurrence rate of cholecystitis (*P* = .561). We conducted a univariate analysis to compare patients with recurrent cholecystitis to those with nonrecurrent cholecystitis in terms of various factors (Table 2). No significant intergroup differences were observed in age, sex, gallstone presence, CCI score, perforation status, CBD stone presence, need for endoscopic retrograde cholangiopancreatography (ERCP), PTGBD catheter indwelling time, WBC count, serum CRP, and gallstone size, and number. However, there were significant differences between the two groups in the presence of cystic duct stones (*P* = .048), catheter migration status (*P* = .025), and serum bilirubin level (*P* = .002). Multivariable analysis with Cox proportional hazards modeling revealed that cystic duct stones, with a hazard ratio (HR) of 4.49, a 95% confidence interval (CI) of 1.37–14.69 (*P* = .01), and catheter migration (HR, 4.45; 95% CI, 1.70–11.65; *P* = .002) were independent risk factors for recurrent cholecystitis after PTGBD (Table 3). The interval between PTGBD catheter removal and cholecystitis recurrence was not shorter in patients with cystic duct stones (n = 4) compared to those with only gallbladder stones (n = 10) (217.5 ± 267.3 days vs 318.9 ± 310.3 days, respectively; *P* = .578).

**Table 2:** Univariate analysis of the clinical factors influencing recurrent cholecystitis.

Clinical factors	Non-recurrent cholecystitis (n = 95)	Recurrent cholecystitis (n = 20)	*P*
Sex, male	57 (60.0)	11 (55.0)	.679
Age, y	72.5 ± 10.7	76.4 ± 10.7	.149
Calculous	63 (66.3)	14 (70.0)	.750
Gallstone	53 (55.8)	14 (70.0)	.241
Size ≥1 cm	12 (22.6)	2 (20.9)	.716
Size <0.5 cm	34 (64.2)	10 (71.4)	.756
Solitary	19 (35.8)	3 (21.4)	.359
Cystic duct stone	5 (5.3)	4 (20.0)	.048
Sludge	21 (22.1)	2 (10.0)	.356
Charlson Comorbidity Index score	4.6 ± 2.1	5.0 ± 1.3	.215
Perforation	11 (11.6)	1 (5.0)	.689
CBD stone	19 (20.0)	2 (10.0)	.523
Combined ERCP	35 (36.8)	6 (30.0)	.561
PTGBD catheter indwelling time, days	141.8 ± 283.9	196.9 ± 294.3	.435
Duration of PTGBD ≥6 wk	73 (76.8)	16 (80.0)	1.000
Follow-up cholangiogram	58 (61.1)	9 (45.0)	.186
Clamping of the PTGBD catheter	36 (37.9)	5 (25.0)	.274
PTGBD catheter migration	20 (21.1)	9 (45.0)	.025
WBC count, cells/μL	13,508.2 ± 6537.5	11,713.0 ± 4272.5	.243
CRP, mg/dL	15.3 ± 9.2	13.6 ± 10.9	.466
Bilirubin, serum, mg/dL	2.1 ± 2.5	1.1 ± 0.7	.002

Data are presented as median (range), number (%), or mean ±  standard deviation. CRP = C-reactive protein, ERCP = endoscopic retrograde cholangiopancreatography, PTGBD = percutaneous transhepatic gallbladder drainage, SD = standard deviation, WBC = white blood cell.

**Table 3:** Multivariable analysis of factors affecting recurrent cholecystitis.

Variable	HR	95% CI	*P*
Presence of cystic duct stones	4.493	1.37–14.69	.013
Catheter migration	4.451	1.70–11.65	.002
Bilirubin, serum	0.691	0.45–1.07	.101

HR = hazard ration, CI = confidence interval.

### 3.4 Rescue treatment

Most cases of recurrent cholecystitis after PTGBD catheter removal were managed with catheter reinsertion (75%, n = 15), 3 cases with PTGBD and scheduled cholecystectomy, and 2 cases with conservative antibiotic treatment. All cases of recurrence were treated without additional complications or further recurrence according to the available follow-up data.

## 4 Discussion

Comorbidities are strongly associated with postoperative morbidity and complications.^[1]^ Besides, the prevalence of comorbidities increased with age. Most of the patients in our study were of advanced age, with an overall mean age of 73.1 ± 10.8 years. During the study period, a total of 3816 patients with a mean age of 55.2 ± 14.9 years underwent surgical cholecystectomy at our institution. Previous studies have shown CCI scores >5 to be an independent factor for in-hospital cholecystectomy-related complications.^[5]^ In our study, the mean CCI score was found to be 4.5 ± 1.9. Therefore, alternative and safe treatment options, such as PTGBD, are needed for elderly patients with cholecystitis.

The pathophysiology of cholecystitis can be classified according to the presence or absence of gallstones. Calculous cholecystitis is primarily caused by bile stasis within the gallbladder because of stones embedded in the cystic duct, a condition that can inflame and damage the gallbladder mucosa. In our study, the presence of cystic duct stones was identified as a significant risk factor for recurrent cholecystitis (HR, 4.49; 95% CI, 1.37–14.69; *P* = .013). However, prolonged fasting, immobility, or hemodynamic instability may cause ischemic and chemical injury to the gallbladder epithelium, leading to acalculous cholecystitis.^[6]^ In a previous study, calculous cholecystitis was found to be more strongly associated with recurrent cholecystitis than with acalculous cholecystitis.^[7]^ In the present study, the majority of patients had high CCI scores and could not undergo cholecystectomy. Thus, the mechanism normally responsible for acalculous cholecystitis might have influenced both patients with calculous cholecystitis and those with acalculous cholecystitis.^[8]^ This may explain the relatively similar recurrence rates observed with calculous and acalculous cholecystitis (*P* = .750).

At the beginning of our study, we were curious about the effect of ERCP with distal bile duct sphincterotomy on the incidence of cholecystitis. We hypothesized that sphincterotomy could decrease CBD pressure, thereby promoting bile passage through the cystic duct and diminishing the risk of recurrent cholecystitis. In contrast, other researchers have suggested that sphincterotomy can provoke ascending cholangitis and consequently increase the incidence of cholecystitis.^[9,10]^ In our study, no difference in the incidence of recurrent cholecystitis was observed between the patients who underwent endoscopic sphincterotomy and those who did not (6/41 cases vs 14/74 cases, respectively; *P* = .561). Since we excluded reactive secondary cholecystitis induced by cholangitis, we concluded that CBD pressure changes or infection may have little impact on the cystic duct.

The optimal duration of PTGBD catheter indwelling has not yet been established. In previous studies, a catheter indwelling duration of >6 weeks and clamping of the catheter before removal have been proposed as important factors for preventing recurrent cholecystitis.^[10]^ In our study, however, the duration of PTGBD catheter indwelling was not associated with cholecystitis recurrence (*P* = .435). Moreover, there was no significant difference in recurrence rates between patients who had their PTGBD catheter in place for <6 weeks and those who had them in place for >6 weeks (76.8% vs 80.0%, respectively; *P* = 1.00). Relief from gallbladder inflammation usually occurs approximately 48 to 72 hours after PTGBD, and hepatocutaneous tract maturation takes place in approximately 2 weeks.^[11,12]^ In the present study, catheter migration (with a rate of 25.2%) was the only late complication that was identified as an independent procedure-related risk factor for cholecystitis recurrence. Therefore, we agree that the duration of PTGBD catheter indwelling should exceed 2 weeks after insertion. In addition, great care should be taken to prevent inadvertent dislodging of the PTGBD catheter. Planned early PTGBD removal after improvement of the patient's symptoms and signs is the most important factor for reducing the risk of recurrent cholecystitis. Further prospective studies are warranted to confirm our conclusions.

Recently, instead of the uncomfortable external gallbladder drainage approach, intraluminal drainage methods have been attempted. Intraluminal approaches can be classified as either transpapillary, ERCP, or transmural, using endoscopic ultrasound (EUS) guidance.^[13,14]^ The main limitation of the former approach is its relatively low success rate (approximately 75%), which results from the difficulty of accessing the cystic duct under fluoroscopic guidance.^[15,16]^ After Baron et al reported successful EUS-guided transduodenal drainage of the gallbladder, further studies focused on using various types of metallic or plastic stents for EUS-guided transmural drainage of the gallbladder.^[17]^ This approach achieved a clinical success rate of >95%.^[18,19]^ In addition, single-operator peroral cholangioscopy (SpyGlass DS; Boston Scientific, Natick, MA) has been reported as a treatment option for patients with cholecystitis.^[20]^ Therefore, for patients at high risk for recurrent cholecystitis, such as those with cystic duct stones or those experiencing slow improvement in their signs and symptoms, intraluminal drainage could be performed as a permanent treatment instead of PTGBD. Additionally, with further development of the instruments and techniques utilized in the endoscopic transluminal approach, PTGBD can be used as the initial treatment of choice for cholecystitis.

There are some limitations to our study. First, it is possible that our retrospective study may have been affected by selection bias. To decrease the possibility of selection bias, it is important to set strict inclusion and exclusion criteria. Therefore, we carefully filtered the PTGBD cases according to the Tokyo guidelines for acute cholecystitis. Additionally, cases of secondary cholecystitis, those involving direct tumor invasion into the cystic duct, reactive cholecystitis caused by other bile duct infections, and cases of ambiguous calculous/acalculous cholecystitis were excluded. Second, we did not include all cases of cholecystitis. In some cases, conservative treatment with intravenous antibiotics was the main form of treatment without surgery or PTGBD. However, most cases treated conservatively were mild cases of cholecystitis. Hence, our study may be worthwhile because it incorporates moderate and severe cases of cholecystitis.

In conclusion, endoscopic biliary sphincterotomy does not reduce post-PTGBD recurrence of cholecystitis; therefore, specific strategies are needed to reduce the incidence of recurrent cholecystitis after PTGBD. Furthermore, reconsideration of surgical cholecystectomy or intraluminal drainage of the gallbladder is recommended for cases involving cystic duct stones. Additionally, when cholecystitis is managed with PTGBD, caution should be exercised not to inadvertently dislocate the PTGBD catheter. Scheduled removal of the catheter according to clinical improvement is also important to help prevent cholecystitis recurrence.

## Acknowledgments

The authors thank KNUH ERCP team nurses/radiological technologists and Dr. Eugene Kwon.

## Author contributions

Jun Heo: data acquisition, data analysis and interpretation, drafting of the manuscript, statistical analysis, and study supervision

Min Kyu Jung: study concept and design, critical revision of the manuscript for important intellectual content, technical and material support, and study supervision

Chang Min Cho: data acquisition and technical and material support

Sang Yub Lee: data acquisition, drafting of the manuscript, and technical and material support

Hun Kyu Ryeom: data acquisition and technical and material support

Jae Min Chun: data acquisition, data analysis and interpretation, and critical revision of the manuscript for important intellectual content

Young Seok Han: data acquisition and technical and material support

Hyung Jun Kwon: data acquisition and technical and material support

**Conceptualization:** Jun Heo, Min Kyu Jung, Chang Min Cho.

**Data curation:** Jun Heo, Min Kyu Jung, Sang Yub Lee, Jae Min Chun, Young Seok Han, Hyung Jun Kwon.

**Formal analysis:** Jun Heo, Hyung Jun Kwon.

**Investigation:** Jae Min Chun, Young Seok Han.

**Project administration:** Chang Min Cho.

**Software:** Hun Kyu Ryeom.

**Supervision:** Min Kyu Jung, Chang Min Cho, Sang Yub Lee, Hun Kyu Ryeom, Jae Min Chun, Young Seok Han.

**Validation:** Min Kyu Jung, Sang Yub Lee, Hun Kyu Ryeom.

**Visualization:** Jun Heo.

**Writing – original draft:** Jun Heo.

**Writing – review & editing:** Min Kyu Jung.
